# Performance evaluation of enhanced SBR in simultaneous removal of nitrogen and phosphorous

**DOI:** 10.1186/s40201-014-0134-2

**Published:** 2014-11-13

**Authors:** Tahereh Jafarzadeh Ghehi, Soheil Mortezaeifar, Mitra Gholami, Roshanak Rezaei Kalantary, Amir Hossein Mahvi

**Affiliations:** Department of Environmental Health Engineering, School of Public Health, Tehran University of Medical Sciences, Tehran, Iran; Department of Environmental Engineering (Water Resources), Science and Research Branch, Faculty of Environment and Energy (FEE), Islamic Azad University, Tehran, Iran; Department of Environmental Engineering, School of Public Health, Iran University of Medical Sciences, P.O.Box:15875–4199, Tehran, Iran; Center for Water Quality Research, Institute for Environmental Research, Tehran University of Medical Sciences, Tehran, Iran

**Keywords:** Biological nutrient removal, Enhanced biological SBR, Phosphorous removal, Nitrification- denitrification, C/N/P ratio

## Abstract

**Background:**

Simultaneous nitrogen, phosphorous and COD removal in a pilot-scale enhanced Sequencing Batch Reactor (eSBR) was investigated.

**Methods:**

The reactor consisted of a pre-anoxic zone and internal recycle and was fed with synthetic wastewater. The study was performed by operating the reactor in 6-hour cycles in three different operational modes during a time frame of 279 days.

**Results:**

Under the best operational conditions, the average removal rate of COD, TN, and TP were obtained as 93.52, 88.31, and 97.56%, respectively.

**Conclusions:**

A significant denitrifying phosphorus removal (more than 80%) occurred at run1 and 3 which started the cycle under anoxic condition.

## Background

In biological wastewater treatment processes, sufficient nutrients are required for bacterial growth and floc formation [1]. However, excess organic matters in the effluents like nitrogen and phosphorus must be removed prior to their discharge into water bodies to prevent eutrophication, oxygen depletion and toxicity. There are some strict criteria for discharging effluents containing nitrogen and phosphorus, especially in environmentally sensitive areas. The stringent discharge limits, have also been established for COD, TP, and bacteriological qualities [2,3].

Incorporation of an anoxic phase permits the combined removal of nitrogen and phosphate from wastewater [4]; therefore, it is desired to remove both N and P through the combined systems [5]. The most recognized and practical wastewater treatment technology is the activated sludge system. Which has been further developed to achieve biological nutrient removal [2]. Biological processes are a cost effective and environmental friendly method compared to chemical treatment method. They minimize the production of waste solids and reduce energy consumption [4,6].

The traditional or conventional biological processes can remove nitrogen efficiently in separate aerobic and anaerobic phases which are generally carried out in separate bioreactors or using different aeration intervals. Sequencing batch reactor (SBR) as an easily obtainable, on time scale, highly operational and flexible technology, is a promising alternative to continuous “Completely Stirred Reactors” [7]. The SBR systems have many advantages such as lower operational cost, less bulking and higher flexibility to combine nitrification and denitrification phases into one reactor and subsequently into a small treatment plant [8]. This process has a good performance for nitrogen, phosphorus and COD removal [9]. Since nutrient removal in a SBR takes place through alternating anaerobic and anoxic/aerobic periods, nitrification, denitrification and phosphorous removal, all happens during the reaction period of SBR within on/off cycles of air/mixers [9].

Biological Nutrient Removal (BNR) is one of the methods that can reduce waste solid production [4]. Nitrogen removal is performed under aerobic and anoxic conditions by *autotrophic nitrifier and heterotrophic denitrifier bacteria* [10]. In simultaneous Nitrification and Denitrification (SND) processes, under reduced aeration, both processes are achieved concurrently, therefore it is not necessary to control the aerobic and anaerobic microbial community [7]. It should be noted that if the influent COD concentration is insufficient, denitrification or phosphorus release would decrease [11], thus the low ratio of biodegradable organic substrate to nitrogen and phosphorus contents is a limiting factor in the biological nitrogen removal. Since the *denitrifying bacteria* compete for carbon sources with other *heterotrophs*, a low carbon to nitrogen ratio in the influent leads to a rapid carbon deficit, causing an unbalanced concurrent nitrification and denitrification [12]. It is concluded that C:N:P ratio is essential for biological nutrient removal.

Phosphorus removal from wastewater can also be achieved by biological or chemical methods [13]. In biological phosphorus removal, *Polyphosphate Accumulating Organisms (PAOs)*, largely responsible for P removal, take up large amounts of phosphate as intracellular polyphosphate from wastewater when they are put under alternating anaerobic and anoxic/aerobic conditions [4,13,14].

Enhanced Biological Phosphorous Removal (EBPR) and Nitrogen removal, take considerable time that must be taken into account, when operating with the minimum sludge recycle ratio. According to Singh M et al., applying anoxic condition right after the aeration period improve the N-removal efficiency, though the external carbon sources such as glucose, methanol, acetate, and propionate are required for the treatment of dilute wastewaters [15].

In current study, simultaneous removal of N and P is investigated in a modified novel SBR, known as enhanced SBR (eSBR). In addition to the advantages of the typical SBR, eSBR can make an efficient use of influent COD as carbon source that is required in denitrification process.

The performance of eSBR with pre-anoxic zone and internal recycle was investigated in 9 scenarios.

## Methods

### Experimental set-up

**Figure 1:**
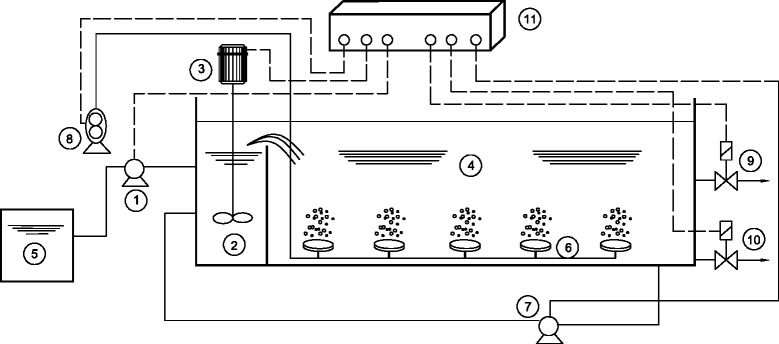
**Schematic diagram of eSBR system and control equipment used in the reactor.** (1) feed pump; (2) pre-anoxic zone; (3) mixer; (4) main zone (5) influent; (6) air diffuser; (7) return activated sludge pump; (8) air pump; (9) effluent solenoid valve; (10) excess sludge valve; (11) mini PLC.

A pilot-scale plexiglass reactor with a working volume of 26 liters was designed and operated in a laboratory, using synthetic wastewater (Figure 1). The eSBR reactor contained a pre-anoxic zone and a main zone that was divided by a wall to buffer the continuous inflow, suppress bulking, foaming, and minimize the short circuiting. According to Ge et al., sludge bulking can be suppressed by setting a selector and alternate between anoxic and oxic conditions [11].

The pre-anoxic zone received the synthetic wastewater continuously. The diffusers that provided aeration and agitation for the mixed liquor in the main zone were connected to an air pump. Return Activated Sludge (RAS) pump was employed to recycle biomass that was transferred from the react zone to pre–anoxic zone during the anoxic period. After phase settlement, the effluent was decanted from the reactor by a solenoid valve, as well as excess sludge that was wasted during the decant phase. The operation of the system was controlled by a Programmable Logic Controller (PLC).

In addition, pH, ORP, and DO were monitored as key parameters during the aerobic and anoxic phases to ensure the desirable performance of the reactor. Samples were also collected daily. Technical specification of the eSBR is given in Table 1.

**Table 1:** **Technical specification for the eSBR reactor**

**Parameter**	**Volume (L)**	**Pre-anoxic/main zone volume (%)**	**Filling volume/total volume (%)**	**Flowrate (L/d)**	**Cycle time (h)**	**Settle duration (min)**	**Decant duration (min)**	**Recycle/inflow (%)**
eSBR	26	10	30	30	6	60	15	30

### Analytical methods

COD, Sludge Volume Index (SVI), Mixed Liquor Suspended Solids (MLSS), Total Nitrogen (TN), Total Kjeldal Nitrogen (TKN), Nitrate(NO_3_-N), Nitrite (NO_2_-N), Total Phosphorous (TP), Carboneous Biological Oxygen Demand (CBOD), and Total suspended solid (TSS,2540B) were measured according to Standard methods [16]. Total Phosphorus was measured using HACH methods (HACH Odyssey DR/2500). Temperature and pH were analyzed by WTW level-2 pH meters (WTW Company, Germany). Oxidation reduction potential (ORP) and dissolved oxygen (DO) were monitored by WTW, pH/oxi340i meter by mean of ORP and DO probes (WTW Company, Germany).

### Wastewater and seed sludge characteristics

The influent used in the lab-scale eSBR was synthetic wastewater which was prepared on a daily basis. The seeding sludge was obtained from Zargandeh Municipal Wastewater Treatment Plant (Tehran, Iran) and it was acclimatized to the synthetic wastewater for 30 days prior to launching of the pilot plant.

The influent with the composition that is shown in Table 2 was used in this study. Carbon, nitrogen and phosphorus were added as glucose, ammonium chloride and monopotassium phosphate at different concentrations to achieve the various desired C:N:P ratios of 100:5:1, 50:5:1 and 25:5:1, 769 mg of sodium acetate (600 mg/L as COD basis), 43.9 mg of KH_2_PO_4_ (10 mg/L as PO_4_-P basis), 229.3 mg of NH_4_Cl (60 mg/L as NH_4_-N basis), 90 mg of MgSO_4__7H_2_O, 14 mg of CaCl_2_ _2H_2_O and 0.3 mL of trace solution per litre. The composition of trace element solution per litre was as follows: 1.5 g of FeCl_3_ _ 6H_2_O, 0.15 g of H_3_BO_3_, 0.03 g of CuSO_4_ _5H_2_O, 0.18 g of KI, 0.12 g of MnCl_2_ _H_2_O, 0.06 g of Na_2_MoO_4_ _2H_2_O, 0.12 g of ZnSO_4_ _ 7H_2_O, 0.15 g of CoCl_2_ _ 6H_2_O and 10 g of EDTA [17]. The MLSS varied from 3,100 to 4,200 mg/L. After achieving the steady state, the experiments were carried out for 9 months. The characteristic of influent wastewater has been listed in Table 2.

**Table 2:** **Characteristics of the synthetic wastewater used during the 279-day operation**

**C:N:P**	**COD (mg/L)**	**TN (mg/L)**	**TP (mg/L)**	**pH**	**Temperature (0C)**
100:5:1	450 ± 60	22.5 ± 2.5	4.5 ± 1	6.5 ± 1.5	21-24
50:5:1	450 ± 60	45 ± 5	9 ± 2	6.5 ± 1.5	21-24
25:5:1	450 ± 60	90 ± 10	18 ± 4	6.5 ± 1.5	21-24

### Operation strategy

**Figure 2:**
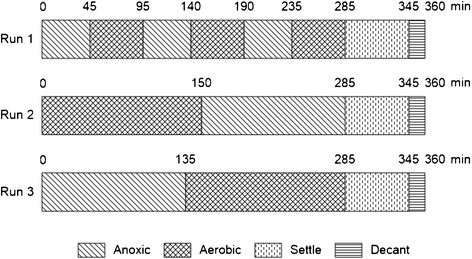
**Operational modes of the eSBR.**

For the continuous eSBR using three 6-hour cycles, the efficiency of different operational modes (Run1, Run2, and Run3) with fill-react, fill-settle, and fill-decant sequences, were all evaluated. Figure 2 demonstrates three operational modes of the pilot.

Removal efficiency of COD, total nitrogen and phosphorous, were all investigated in three operational modes and variable C:N:P ratios. The fill react sequence in Run1 consisted of three sequential anoxic/aerobic phases; while, Run2 and 3 only included one aerobic and anoxic period. The activated sludge was recycled during anoxic period, and the resulting sludge was wasted during the decant phase to keep the MLSS concentration at a certain level.

## Results and discussion

Comprehensive results of the eSBR under various C:N:P ratios and the operating conditions are given in Table 3.

**Table 3:** **The eSBR performance in various operational modes and influent ratios**

**Operational modes**		**Run 1**			**Run 2**			**Run3**		
C:N:P		(100:5:1)	(50:5:1)	(25:5:1)	(100:5:1)	(50:5:1)	(25:5:1)	(100:5:1)	(50:5:1)	(25:5:1)
Removal efficiency (average)	COD (%)	93.52	93.94	94.35	89.96	94.15	94.31	93.77	93.99	94.27
	TN (%)	88.31	83.49	67.88	78.09	72.82	59.34	78.44	78.07	66.34
	TP (%)	97.56	81.89	27.72	57.11	60.22	16.61	96.89	90.67	22.72

### COD removal

**Figure 3:**
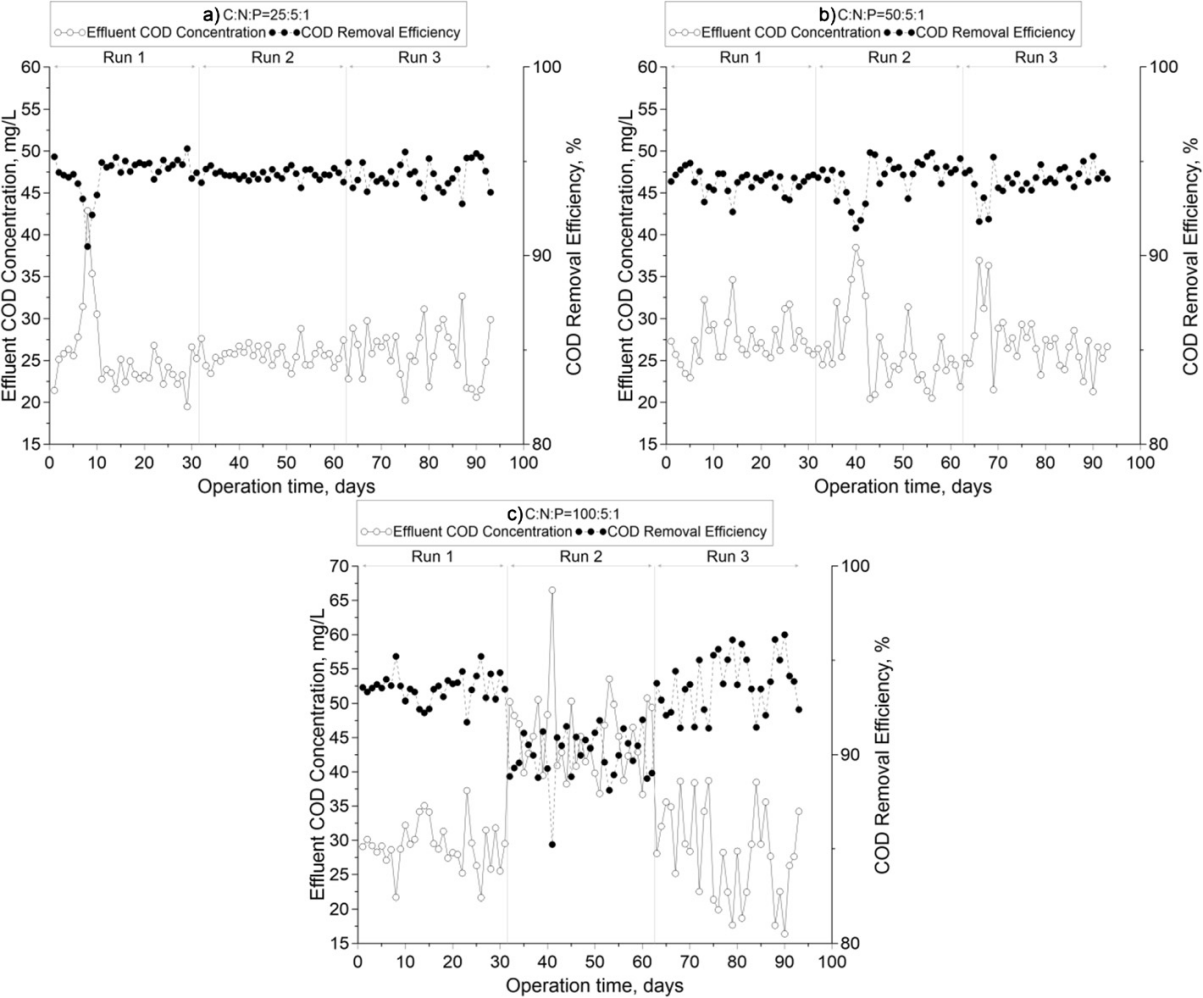
**Time course profiles of the COD removal: (a) 25:5:1, (b) 50:5:1and (c) 100:5:1 ratios.**

The time course profile and percentage of COD removal under various C:N:P ratios and operating conditions are all shown in Figure 3. All COD removal efficiencies were found to be almost the same (approximately 94%), regardless of the operational condition and C:N:P ratio. However, the effluent COD at the C:N:P ratio of 25:5:1 was slightly lower than those of 100:5:1 and 50:5:1. Therefore- it seems that in all the studied ratios, nitrogen was in excess in carbon metabolism process; whilst, in other study [1] the complete carbon removal was achieved at the C:N:P ratio of 100:1.9:0.5. Tian et al., reported 90% COD removal efficiency in a bench scale EBPR reactor in steady state condition [18].

### Nitrogen removal

**Figure 4:**
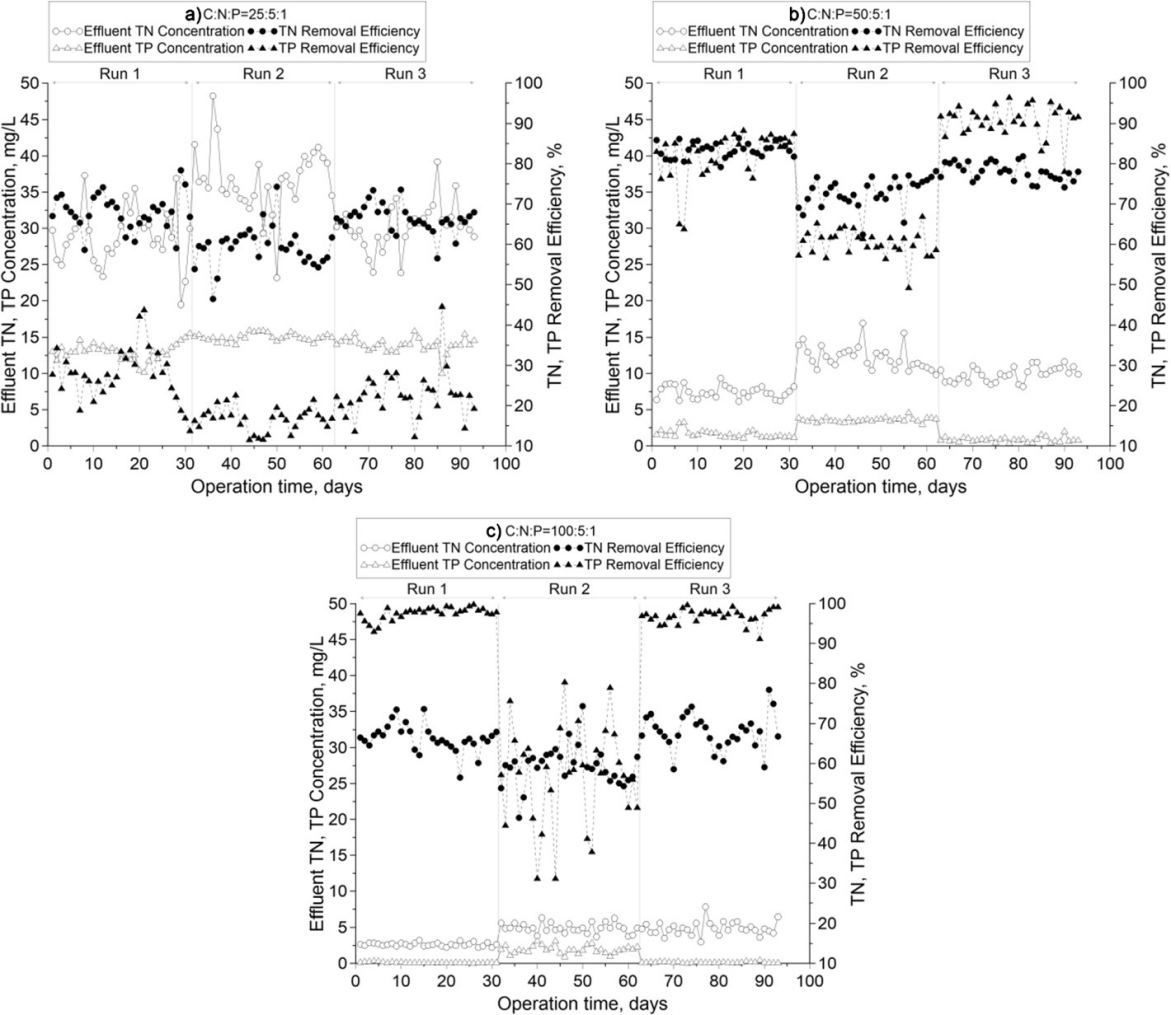
**Time course profiles of total-N and total-P removal at: (a) 25:5:1, (b) 50:5:1and (c) 100:5:1 ratios.**

The time course profile and percentage of TN removal under various C:N:P ratios and operating conditions are shown in Figure 4.

In Run1, the TN average removal efficiencies at 100:5:1, 50:5:1 and 25:5:1 ratios were 88.31, 83.49 and 67.88%, respectively. On the second 31 days in Run2, the average removal efficiencies of TN at the C:N:P ratios of 100:5:1, 50:5:1 and 25:5:1, were 78.09, 72.82 and 59.34%, respectively. In Run3 from the 62nd to 93rd day, the average removal efficiencies of TN at the C:N:P ratios of 100:5:1, 50:5:1 and 25:5:1 were 78.44, 78.07 and 66.34%, respectively (Table 3); While in a moving bed biofilm reactor (MBBR) which was studied by Kermani et al., nitrogen removal efficiency of 80.9% was reported in COD/NH_4_-N ratio of 500/62.5, the result of the MBBR system was almost near to the present study [3]. According to the results, more TN was discharged at the lowest C:N:P ratio (25:5:1). Ge et al., reported high nitrogen removal efficiency (89%) in a modified step feed process when COD/TN ratio was 7.41 [11]; therefore, it seems that low TN removal efficiency in ratio of 25:5:1 is due to low carbon source compared to influent TN concentration. According to Blackburne, COD to TKN ratio of about 5–6 mgCOD/mgN is difficult nature of a domestic wastewater for full nitrogen removal [19].

In a further related study, Kim et al., confirmed that maintaining the low carbon to nitrogen ratio in the influent leads to a rapid carbon deficit in the reactor that would lead to an unbalanced simultaneous nitrification and denitrification [12].

The results also showed that the arrangement of aerobic and anoxic phases greatly affected the TN removal efficiency. As it is shown in Table 3, the highest TN removal efficiency was obtained in Run1 in all C:N:P ratios, implying that an increase in the number of sequences improves the N-removal efficiency. In Run1 and 3, the TN removal efficiency was significantly different from that of Run 2. According to Lee et al., organic acids were produced at the beginning of the cycle that was initiated by an anoxic phase and consequently facilitated denitrification [20].

### Phosphorus removal

The time course profiles and percentage of TP removal under various C:N:P ratios and operating conditions are shown in Figure 4.

In Run1, the TP average removal efficiencies at the 100:5:1, 50:5:1 and 25:5:1 ratios were 97.56, 81.89, and 27.72%, respectively. On the second 31 days in Run2, the average removal efficiencies at the C:N:P ratios of 100:5:1, 50:5:1 and 25:5:1were 57.11, 60.22, and 16.61%, respectively. In Run3, from the 62nd to 93rd day, the average removal efficiencies of TP at the carbon to nitrogen ratios of 100:5:1, 50:5:1, and 25:5:1 were 96.89, 90.67, and 22.72%, respectively (Table 3). Ge et al. evaluated the performance of a pilot scale modified step feed process and reported that a higher level of TP removal efficiency was achieved in COD:P ratio between 35.9 and 92.5 [11]. Therefore it can be concluded that the low ratio of COD:P (25:1) in the current study was the main reason for insufficient removal efficiency (22.72%) of phosphorus.

Run1 and 3 showed the highest average removal efficiency of the total Phosphorus at various influent concentrations (Table 3). In these two mentioned operational modes, alternating anoxic/aerobic phase(s) resulted in high phosphorus removal efficiency compared to Run 2 with an aerobic/anoxic time period during the reaction phase.

PAOs used COD as a primary source of volatile fatty acids [21] and, the conversion of COD to VFAs occurred in the absence of soluble oxygen [22]. Under aerobic conditions, VFAs induced *PAOs* to take up more acids and release phosphorus into the solution [4]. Under the subsequent aerobic condition, the luxury uptake of phosphorous occurred due to oxidation of intracellular polyphosphate led by releasing energy in the form of poly –P bonds into bacteria cell. The high performance of P-removal can be achieved by withdrawing the activated sludge with high poly-p content [3,4]. According to Fu et al., it is reasonable to assume that the internal recirculation of sludge between anoxic and oxic zone induces PAOs’ accumulation [10].

In the current study, doubling TN content of influent didn’t have any significant effect on effluent TN and TP concentration. However, as TN content was increased four folds, the removal efficiency was dramatically decreased (Table 3). Based on previous studies [11], the decrease in TP and TN removal efficiencies might be due to lack of organic substrate for denitrification or phosphorus removal. Therefore, it seems that the EBPR process requires more carbon sources to remove phosphorus from wastewater. Similarly, Slade A et al., observed less effluent phosphorus at the C:N:P ratio of 100:4.9:0.5 than that of 100:0:0.5 under the same influent phosphorus and BOD removal conditions [1].

**Figure 5:**
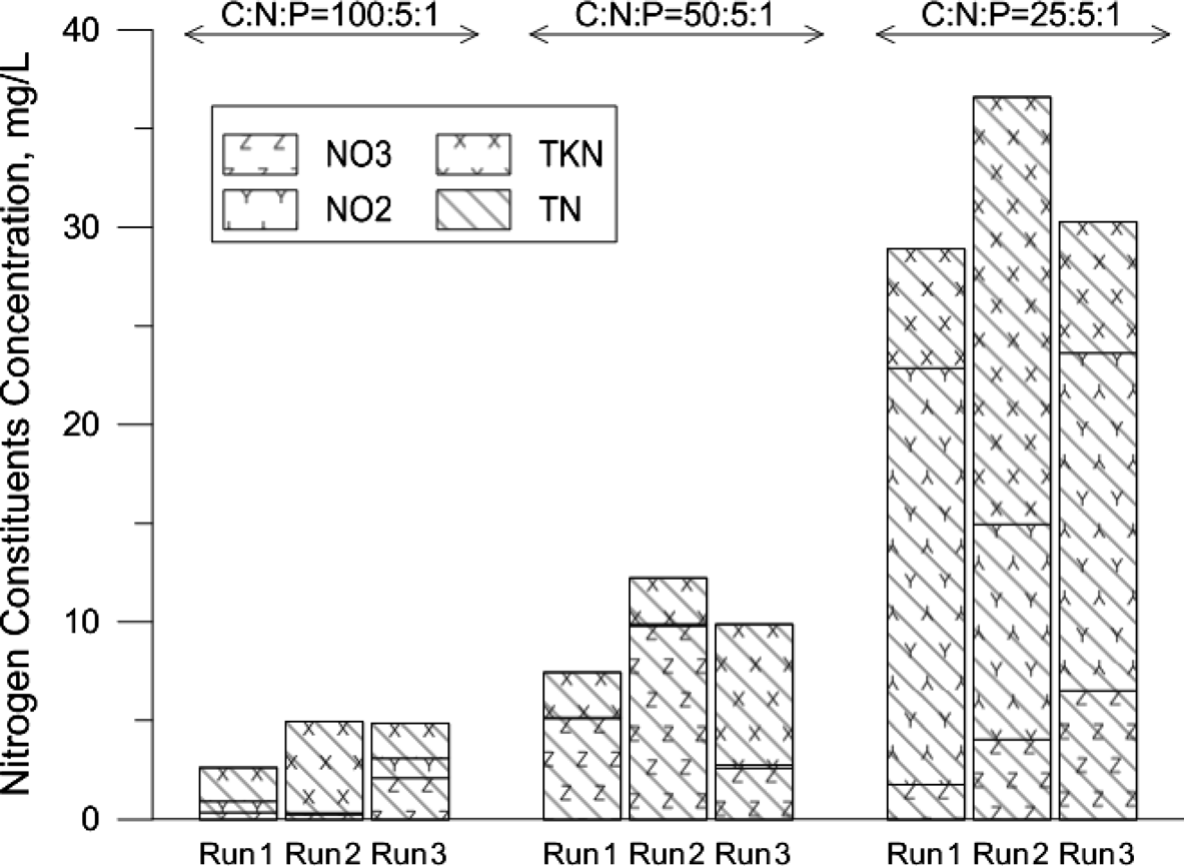
**Nitrogen constituent in the effluent in all operational modes and C:N:P ratios.**

As can be seen in Figure 5, the effluent nitrate at the ratio of 25:5:1 showed the greatest discharge level. The presence of nitrite in the reactor inhibited the aerobic and anoxic phosphorus uptake [14]. Production and accumulation of nitrite in the anoxic phase coincided with increasing the *competibacteria* population that may overcome *GAOs* to *PAOs bacteria*. Oehmen et al. also reported that the PAOs growth rate was inhibited in the presence of nitrite that favors the growth of *GAOs* [14]. In the integrated N & P removal system, high concentration of nitrite acts as a severe inhibitor on wide range of microorganisms that can reduce or eliminate microbial activities [18].

## Conclusion

In this study, simultaneous nitrogen, phosphorus and COD removal performance in an eSBR reactor was investigated. The experimental work lasted for 279 days. The feed was synthetic wastewater with various nitrogen and phosphorus contents. Under several nutrient regimes (C:N:P of 100:5:1, 50:5:1 and 25:5:1) and the various operational conditions no difference was observed regarding the COD removal efficiency. The optimum C:N:P ratio for simultaneous TN and TP removal was found to be 100:5:1, in which the efficiencies of 88.31%, and 97.56% were achieved respectively. The superior performance of the eSBR reactor was due to the application of anoxic pre-zone and sludge recycle that promoted the rapid uptake of soluble substrate. As a conclusion, these findings indicate that the eSBR performance is improved by increasing the number of anoxic/oxic periods. Finally, the present study provides a good basis for the application of eSBR in the future.
